# The role of laser interstitial thermal therapy in enhancing progression-free survival of difficult-to-access high-grade gliomas: a multicenter study

**DOI:** 10.1002/cam4.266

**Published:** 2014-05-09

**Authors:** Alireza M Mohammadi, Ammar H Hawasli, Analiz Rodriguez, Jason L Schroeder, Adrian W Laxton, Paul Elson, Stephen B Tatter, Gene H Barnett, Eric C Leuthardt

**Affiliations:** 1 The Rose Ella Burkhardt Brain Tumor and Neuro-Oncology Center, Department of Neurosurgery, Neurological Institute9500 Euclid Avenue, S70, Cleveland Clinic, Cleveland, Ohio, 44195; 2 Department of Neurosurgery, Washington University School of Medicine660 S. Euclid Avenue, Campus Box 8057, St. Louis, Missouri, 63110; 3 Department of Neurosurgery, Wake Forest School of MedicineMedical Center Blvd, Winston-Salem, North Carolina, 27157; 4 Department of Quantitative Health Sciences, Cleveland Clinic9500 Euclid Avenue, JJN3, Cleveland, Ohio, 44195; 5 Department of Biomedical Engineering, Center for Innovation in Neuroscience and Technology, Washington University School of Medicine660 S. Euclid, Avenue Campus Box 8057, St. Louis, Missouri, 63110; 6 Department of Mechanical Engineering and Material Science, Center for Innovation in Neuroscience and Technology, Washington University School of Medicine660 S. Euclid, Avenue Campus Box 8057, St. Louis, Missouri, 63110

**Keywords:** Anaplastic glioma, GBM, laser ablation, LITT, NeuroBlate System

## Abstract

Surgical extent-of-resection has been shown to have an impact on high-grade glioma (HGG) outcomes; however, complete resection is rarely achievable in difficult-to-access (DTA) tumors. Controlled thermal damage to the tumor may have the same impact in DTA-HGGs. We report our multicenter results of laser interstitial thermal therapy (LITT) in DTA-HGGs. We retrospectively reviewed 34 consecutive DTA-HGG patients (24 glioblastoma, 10 anaplastic) who underwent LITT at Cleveland Clinic, Washington University, and Wake Forest University (May 2011–December 2012) using the NeuroBlate® System. The extent of thermal damage was determined using thermal damage threshold (TDT) lines: yellow TDT line (43°C for 2 min) and blue TDT line (43°C for 10 min). Volumetric analysis was performed to determine the extent-of-coverage of tumor volume by TDT lines. Patient outcomes were evaluated statistically. LITT was delivered as upfront in 19 and delivered as salvage in 16 cases. After 7.2 months of follow-up, 71% of cases demonstrated progression and 34% died. The median overall survival (OS) for the cohort was not reached; however, the 1-year estimate of OS was 68 ± 9%. Median progression-free survival (PFS) was 5.1 months. Thirteen cases who met the following two criteria—(1) <0.05 cm^3^ tumor volume not covered by the yellow TDT line and (2) <1.5 cm^3^ additional tumor volume not covered by the blue TDT line—had better PFS than the other 21 cases (9.7 vs. 4.6 months; *P* = 0.02). LITT can be used effectively for treatment of DTA-HGGs. More complete coverage of tumor by TDT lines improves PFS which can be translated as the extent of resection concept for surgery.

## Introduction

Treatment of high-grade glioma (HGG) remains a challenge 1. Because of their diffuse nature and infiltration into the surrounding normal brain tissue, complete elimination of tumor cells is not achievable by the use of focal treatment modalities like surgery 2. Resultantly, postoperative treatments such as radiation therapy, and chemotherapy, are indicated in most cases after surgery 3–5. Despite this, a growing body of evidence has shown that aggressive surgical treatment as an upfront cytoreductive procedure has an impact on outcomes for high-grade glioma patients 6–10. Without initial cytoreductive treatment, radiation and chemotherapy have more limited efficacy 11,12. Unfortunately, in difficult-to-access (DTA) tumors, aggressive surgical resection is often not feasible 13. Additionally, with regard to recurrent malignant glioma, the efficacy of available treatment options is limited 14,15. Hence, a need for a new cytoreductive treatment modality exists.

Laser-induced hyperthermia bears merit for consideration as a cytoreductive treatment option. In 1983, Bown 16 showed that for lasers with greater tissue penetration (e.g., Nd-YAG) a wider range of therapeutic effects are seen due to tissue hyperthermia. Subsequently laser-induced thermal therapy was used as treatment for multiple different brain pathologies 17–19. Despite early enthusiasm for this technology, this treatment modality failed to be widely accepted as a therapy for glioma patients, due, in part, to limitations in monitoring the extent of thermal damage delivered during treatment 13,20–24. Early on, various techniques were used to define the extent of thermal damage induced by lasing 19,21,25,26. Eventually, MR-thermography based on the temperature dependence of the proton resonance frequency (PRF) was used to provide real-time image guidance of the extent of thermal damage from lasing 27. Such advances in technology are responsible for the resurgence in laser interstitial thermal therapy (LITT) in recent years 13,28. The NeuroBlate® System (Monteris Medical Corporation, Plymouth, MN) is one of the first LITT devices developed in the modern MR-thermography era. After a successful first in humans study 13, FDA 510k clearance (K081509) was received in May 2009 without any specific limitations for intracranial use.

In this study, we report our multicenter series of LITT for treatment of high-grade glioma patients with the NeuroBlate System. Volumetric analysis was performed to evaluate the extent of thermal damage to tumor tissue and its impact on patient outcomes.

## Methods

### Study design, size, and setting

This is a retrospective multicenter review of high-grade glioma patients who were treated with the NeuroBlate System. Thirty-four consecutive glioblastoma (GBM) and anaplastic glioma patients who underwent a total of 35 LITT procedures from May 2011 to December 2012 at the Cleveland Clinic (Cleveland, OH), Barnes-Jewish Hospital (Washington University, St. Louis, MO), and Wake-Forest Hospital (Winston-Salem, NC) were included. The eligibility criteria included all patients who were greater than 18 years and had pathology proven high-grade glioma (anaplastic glioma or GBM). One patient was excluded from the study—he had a history of prior GBM with multiple prior treatments including radiation therapy and his pre-LITT biopsy showed necrosis and no evidence of recurrent glioma. Patients underwent appropriate postoperative adjuvant treatment and were followed with serial MR imaging every 3 months. The median patient follow-up was 7.2 months (range: 0.1–23 months).

### Data source

Multiple data sources were used for collecting the data. Demographic data, patient characteristics, and outcomes were collected by reviewing the patient charts after Institutional Review Board approval at each center. Volumetric analysis of tumor coverage by thermal damage threshold lines (TDT lines) was performed by importing the pre-, intra-, and postoperative MRI scans as well as the TDT lines into iPlan software (Brainlab, Munich, Germany).

### Procedure

**Figure 1:**
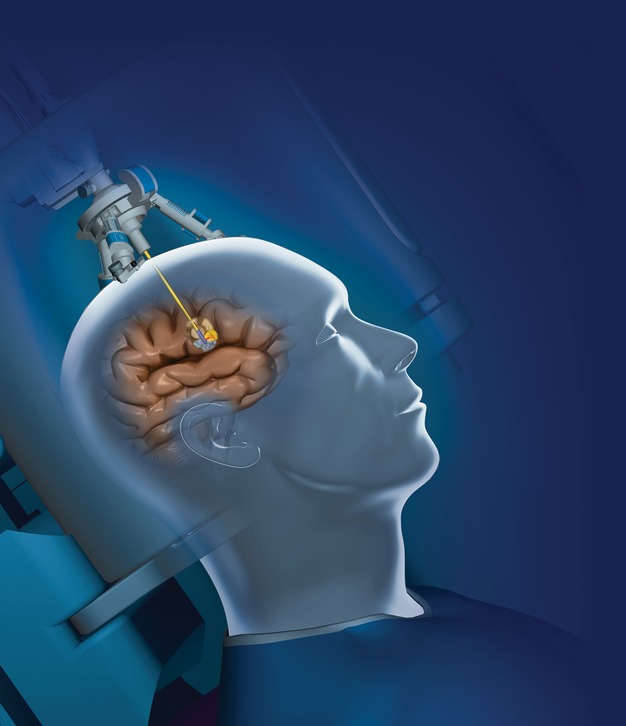
Schematic picture of NeuroBlate System for treatment of difficult to access brain tumors.

The LITT procedure was performed with the NeuroBlate System which uses a solid state diode laser in the Nd-YAG range (1064 nm at 12 W). This laser energy is transferred to the target tissue via a CO_2_ gas-cooled side-firing (directional) laser probe (Fig.1). The trajectory planning and insertion of the laser probe into the tumor were completed through the use of surgical navigation devices and a variety of tools specific to the NeuroBlate System. The location of the laser probe within the tumor was confirmed by intraoperative MRI. The lasing portion of the treatment was planned and controlled via the NeuroBlate System computer workstation utilizing proprietary M°Vision™ (Monteris Medical Corporation, Plymouth, MN) software under real-time MR-thermography guidance. The real-time extent of thermal ablation is calculated by the M°Vision software based on the algorithm of heat kill of cells (a relationship between time and temperature) and demonstrated as thermal damage threshold (TDT) lines which include distinct yellow and blue TDT lines. The yellow TDT line encircles the area of tissue that has been heated to the equivalent of 43°C for at least 2 min and the blue TDT line encircles the area of tissue that has been heated to 43°C for at least 10 min or heated to a higher temperature for a shorter interval 13.

### Variables

**Figure 2:**
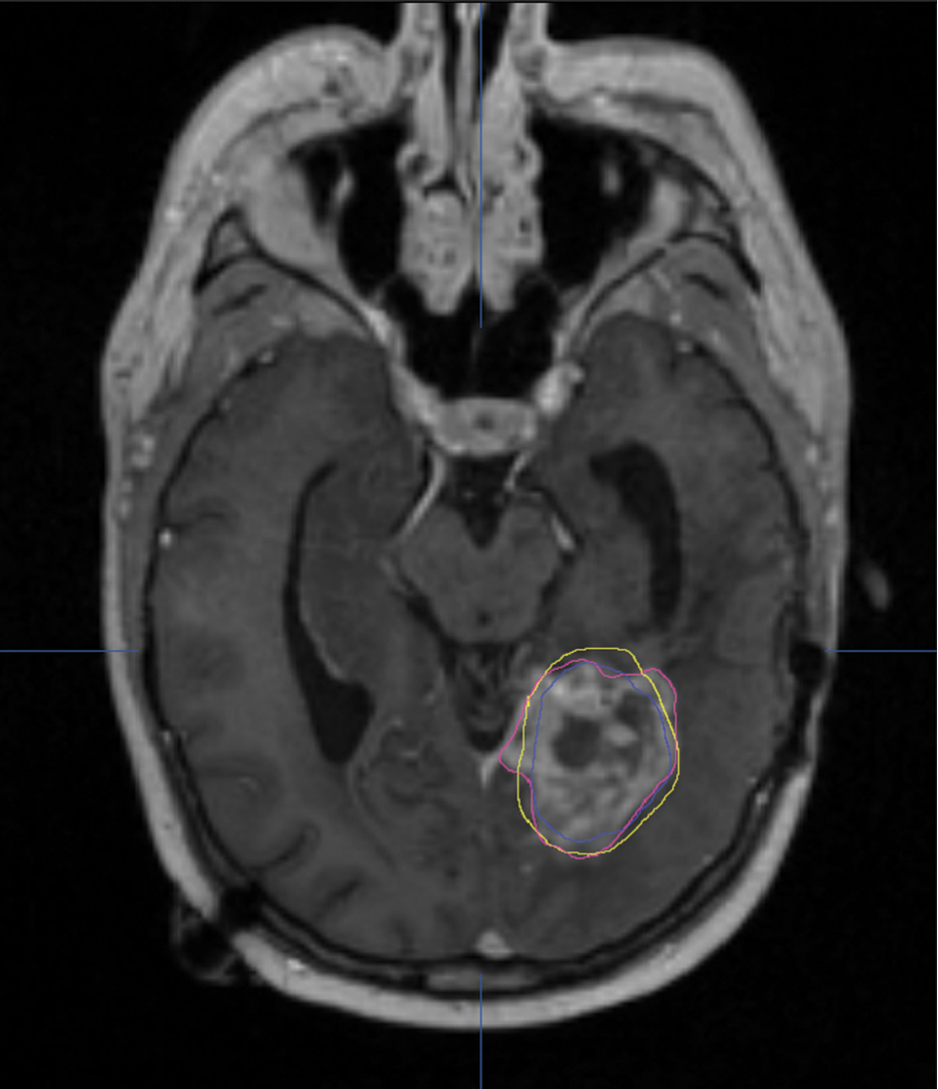
Blue and yellow TDT lines as well as tumor outline (pink) were imported to iPlan software for volumetric analysis.

Progression-free survival (PFS) was chosen as the primary end point of the study. Progression was defined as an increase in thickness of enhancement in a previously treated enhancing lesion, new enhancement, and/or an increase in the cerebral blood volume (CBV) on MR-perfusion sequences; and PFS was measured from the date treatment started to the date of documented progression or death from neurological causes, whichever came first. Secondary study end points were overall survival (OS) and rate of surgical complications. The extent of tumor coverage (percentage of the tumor volume encircled) by the blue and yellow TDT lines as well as the residual tumor volume (volume of tumor tissue not encircled) by each of the TDT lines were measured using iPlan software (Fig.2) and evaluated as possible predictive factors for PFS and OS. These results were also considered within the context of other potential prognostic factors such as gender, age, preoperative Karnofsky performance status (KPS), preoperative neurological function status (NFS), tumor pathology (anaplastic vs. GBM), type of treatment (upfront vs. salvage), adjuvant treatment after LITT, and tumor volume to try to adjust for possible confounding.

### Statistical method

Patient characteristics were summarized using frequency counts and percentages for categorical factors and medians and ranges for continuous factors. PFS and OS were summarized using the Kaplan–Meier method. For convenience a recursive partitioning algorithm was used to identify cutpoints for the extent of coverage defined by the blue and yellow TDT lines and residual volumes. Spearman rank correlations were used to assess the relationship between tumor coverage by the blue and yellow TDT lines and overall tumor volume. The log-rank test and proportional hazards models were used for univariable and multivariable comparisons of PFS and OS. SAS version 9.2 (SAS Institute Inc., Cary, NC) was used for all data analyses.

## Results

### Participants

Among more than 60 patients who underwent the NeuroBlate procedure at all three medical centers during the study period, 34 patients had the diagnosis of high-grade glioma.

### Descriptive data

Median age at the time of LITT was 56 years (range: 19–79 years) and 13 (38%) patients were female. Twenty-four patients were diagnosed with GBM, six patients with anaplastic astrocytoma (AA), and four patients with anaplastic oligodendroglioma (AO). Among 34 patients, 16 procedures (in 16 patients) were performed as upfront treatment for newly diagnosed HGG and 19 procedures (in 18 patients) were performed for treatment of recurrent disease. In terms of tumor location, 15 patients had tumors located in frontal lobe, seven tumors were in the thalamic region, five cases each had parietal and temporal lobe tumors, two cases were insular gliomas, and there was one corpus callosum lesion. Median preoperative (i.e., pre-LITT) KPS was 80 (range: 50–90). Median maximum tumor diameter was 3 cm (range: 1.3–5.5 cm) and median tumor volume was 10.13 cm^3^ (range: 0.7–49.9 cm^3^).

Median coverage of tumor volume by the blue TDT line was 91% (range: 28–100%) and by the yellow TDT line was 98% (range: 34–100%). The median residual tumor volume (not covered) by the blue TDT line was 0.66 cm^3^ (range: 0–22.5 cm^3^) and by the yellow TDT line was 0.19 cm^3^ (range: 0–15.5 cm^3^). LITT treatment was performed using 1 (54%) trajectory in 19 cases, 2 (40%) trajectories in 14 cases, and 3 (6%) trajectories in two cases. The median estimated blood loss during surgery was 30 cc (range: 10–100 cc), median postoperative pain score during the first 24 h was 2/10 (data were available in 31 patients), and median postoperative hospital stay was 3 days (range: 1–29 days). A total of 28/35 cases (80%) received adjuvant chemotherapy starting at a median of 3 weeks after surgery. First-line chemotherapy in all newly diagnosed tumor patients was temozolomide. For patients with recurrent disease a wider variety of chemotherapeutic drugs were used including temozolomide in six cases, cytoxan in three cases, bevacizumab and lumostine each in two cases, and procarbazine in one patient.

### Outcome data

Overall 71% (25/35) of cases progressed during follow-up. The estimated median PFS for the cohort is 5.1 months. Among the 25 cases with progression, radiographic progression was diagnosed as a result of new enhancement in 17 cases and increased CBV on perfusion MRI in six cases. Two additional patients died before their first follow-up MRI without clear non-neurological causes and were counted as progression. In the 23 cases showing radiographic evidence of progression, five (22%) cases had local recurrences in the central area of the treatment field, 12 (52%) cases had recurrences at the peripheral region of the treatment field, five (22%) cases had recurrences outside the primary enhancing tumor volume but within 2 cm of the treatment field, and one (4%) patient had remote recurrence in the contralateral cerebral hemisphere.

The median OS had not been reached at the time of analysis, however, 1-year survival is estimated to be 68 ± 9%. Overall, 35% (12/34) of the patients have died—progression was the cause of death in 10 patients and two patients died because of other reasons. When evaluated in terms of pathology, 11/24 patients with GBM and 1/6 patients with AA died during the follow-up period. None of the four AO patients died during follow-up.

### Main results

**Figure 3:**
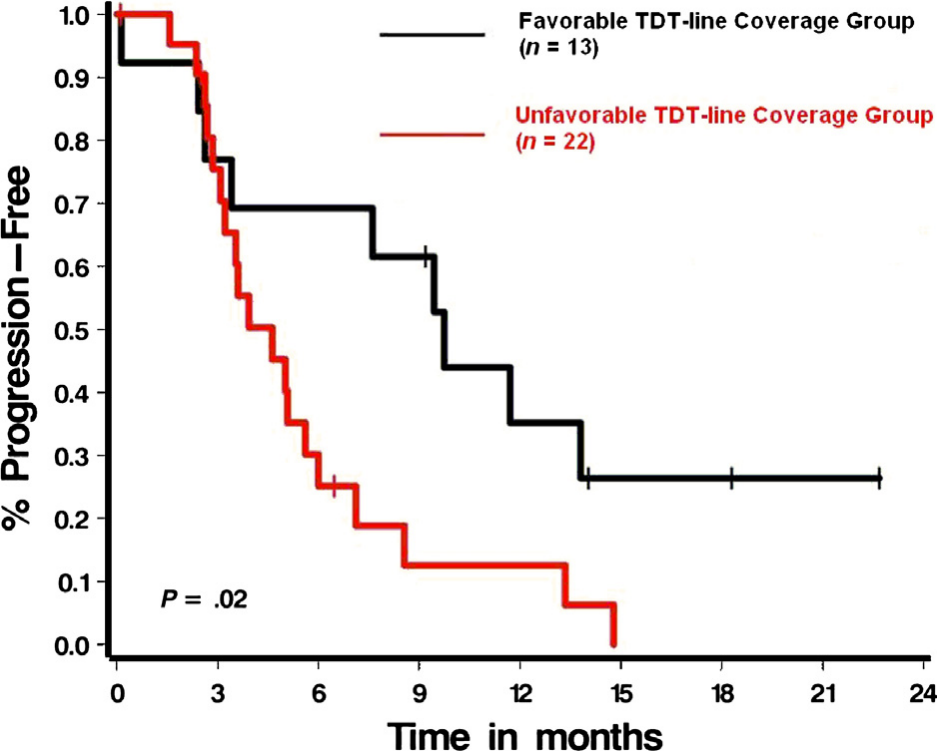
Effect of TDT-line prognostic group on progression-free survival.

Table1 summarizes univariable analyses of PFS. We analyzed volumetric data consisting of blue and yellow TDT lines and overall tumor volume for each patient in the cohort in an effort to determine if there were any characteristics of the TDT lines that were prognostic for PFS and/or OS. Additionally, we analyzed the amount of tumor volume that was covered by the yellow but missed by the blue TDT line as another potential prognostic factor. Cases in which the yellow TDT line missed <0.05 cm^3^ of tumor had a more favorable prognosis (median PFS of 9.7 vs. 4.6 months, *P* = 0.02) as did cases in which the coverage zone between the blue and yellow TDT lines was <1.5 cm^3^ (median PFS of 7.6 vs. 3.3 months, *P* = 0.01). As it was impossible to determine which TDT line is the primary prognostic factor for outcome based on the small size of our sample and the fact that most of the patients with favorable outcomes are common between the two groups, we decided to make a prognostic group based on the combination of blue and yellow TDT line. Hence two groups that were prognostic for PFS could be defined—a favorable group and an unfavorable group. Thirteen cases were included in the favorable TDT line coverage group (defined as tumor volume <0.05 cm^3^ missed by the yellow TDT line and <1.5 cm^3^ of tumor volume covered by the yellow TDT line but uncovered by the blue TDT line) and 22 cases were included in the unfavorable TDT line coverage group (≥0.05 cm^3^ tumor volume missed by the yellow TDT line and/or tumor volume ≥1.5 cm^3^ covered in the transition zone between the yellow and blue TDT lines). The median PFS in the favorable TDT-line coverage group was 9.7 months compared with 4.6 months for the unfavorable TDT-line coverage group (*P* = 0.02) (Fig.3).

**Table 1:** Univariable analysis of progression-free survival.

Factor	Number of patients (%)	Median PFS (months)	*P*-value
Gender			0.92
Female	14 (40%)	5.0	
Male	21 (60%)	5.1	
Age at procedure			0.12
<60 years	19 (54%)	8.6	
≥60 years	16 (46%)	4.5	
Prior radiation/chemotherapy			0.53
No	17 (49%)	3.5	
Yes	18 (51%)	5.8	
Recurrent disease			0.70
No	16 (46%)	3.5	
Yes	19 (54%)	6.0	
Pathology			0.81
Anaplastic glioma	11 (31%)	5.6	
Glioblastoma	24 (69%)	5.1	
TDT-line coverage group			0.02
Favorable	13 (37%)	9.7	
Unfavorable	22 (63%)	4.6	
Location			0.41
Frontal, temporal, parietal lobe	25 (71%)	5.6	
Insula, thalamus, corpus callosum	10 (29%)	3.2	
Tumor volume			0.04
≤10 cm^3^	17 (49%)	8.6	
>10 cm^3^	18 (51%)	4.6	
Preoperative KPS			0.004
90	15 (43%)	7.6	
80 or less	20 (57%)	3.5	
Preoperative neurological function status			0.93
Asymptomatic	11 (35%)	5.8	
Symptomatic	20 (65%)	4.5	
Intracranial hemorrhage (any size)			0.47
No	7 (26%)	6.3	
Yes	20 (74%)	6.0	
Postoperative adjuvant treatment			0.24
No	6 (18%)	5.5	
Radiation or chemotherapy	14 (42%)	5.1	
Radiation and chemotherapy	13 (39%)	9.2	

**Figure 4:**
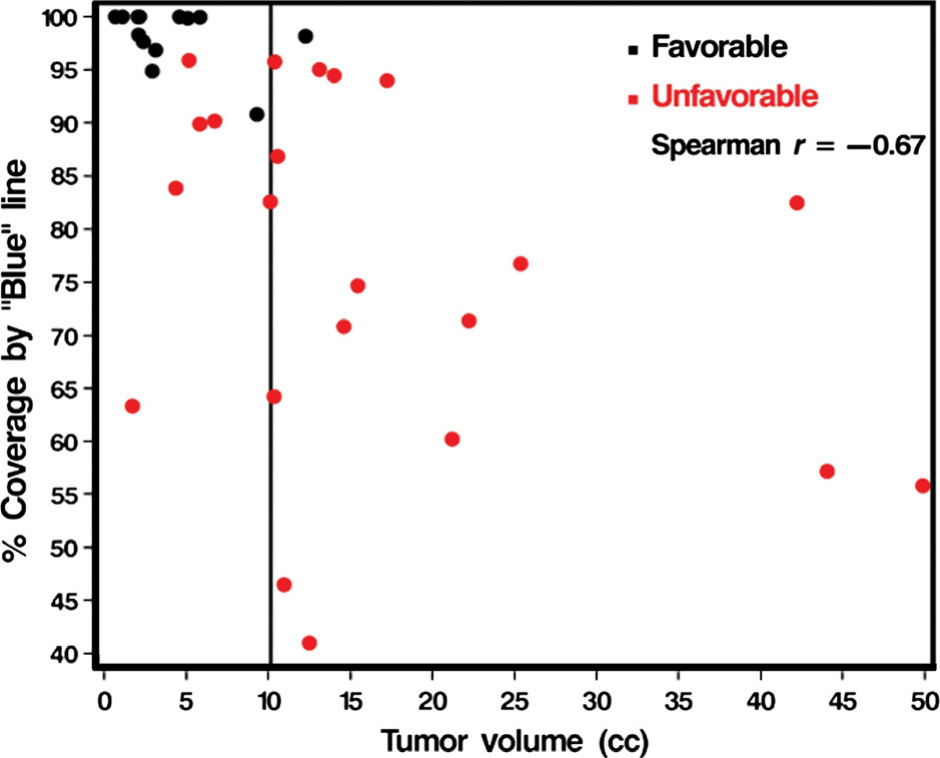
Correlation between tumor volume and the proportion of tumor covered by the “blue” line (for illustration purposes only the “blue” line is considered, but the same holds for the “yellow” line also).

**Figure 5:**
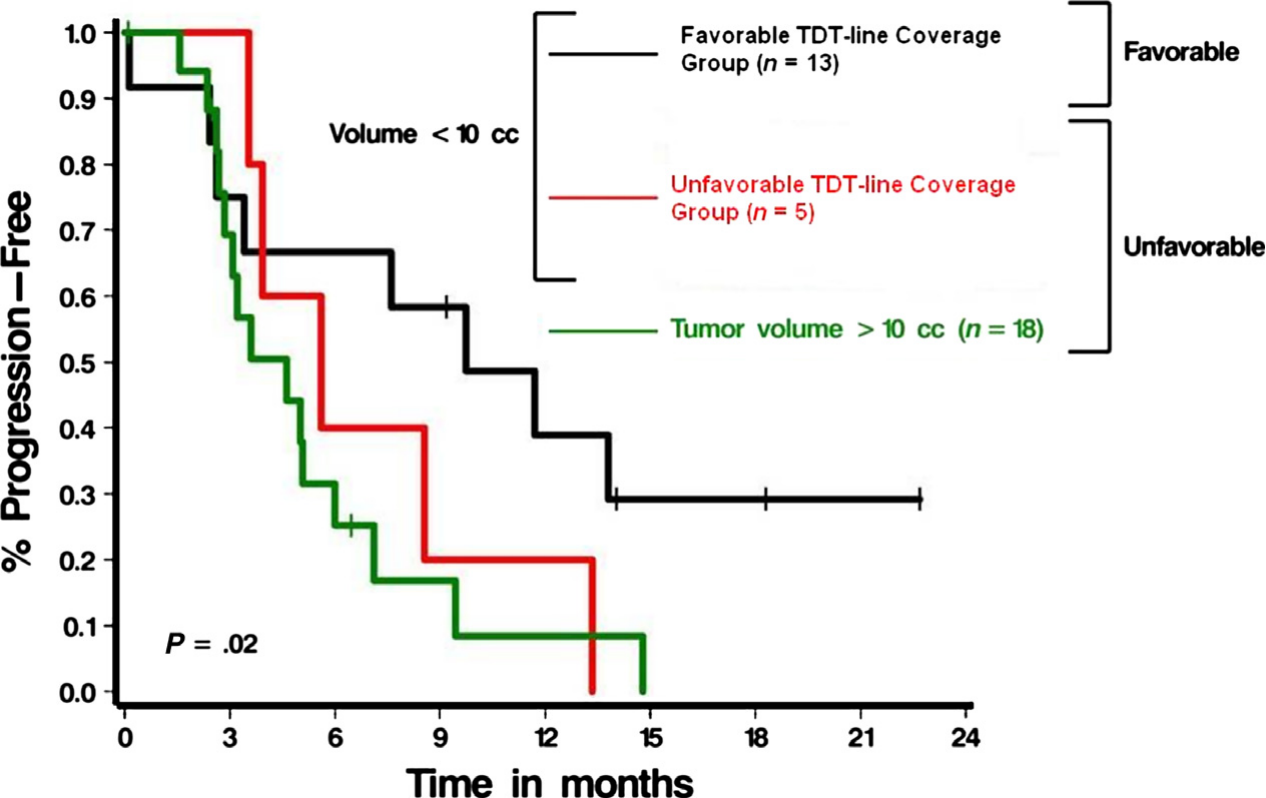
NeuroBlate prognostic groups and tumor volume.

Multivariable models were used to assess the significance of the TDT-line coverage groups adjusting for the possible confounding factors summarized in Table1. Table2 summarizes the results of these analyses and indicates that with the exception of overall tumor volume the TDT prognostic groups remained significantly associated with PFS even after adjusting for other factors. Overall tumor volume is inversely correlated with tumor coverage by the blue and yellow TDT lines as shown in Figure4 (Spearman correlations of −0.67 and −0.66, respectively, both *P*-values <0.0001). It is not surprising therefore that controlling for tumor volume, the effect of TDT prognostic group was marginal (*P* = 0.16). However, as shown in Figure5 the TDT prognostic groups essentially separate out the patients with low-volume disease who have a poorer prognosis from those with a better prognosis (*P* = 0.02).

**Table 2:** Effect on progression-free survival of prognostic groups based on NeuroBlate parameters adjusted for other factors.

Factor	Hazard ratio (95% CI)	*P*-value
TDT-line coverage group (unfavorable vs. favorable)	2.57 (1.12–5.92)	0.03
Gender (female vs. male)	1.02 (0.48–2.17)	0.96
TDT-line coverage group (unfavorable vs. favorable)	3.13 (1.30–7.55)	0.01
Age (≥60 vs. <60)	2.27 (1.02–5.04)	0.04
TDT-line coverage group (unfavorable vs. favorable)	2.90 (1.22–6.90)	0.02
Prior treatment (RT and/or chemo vs. none)	1.62 (0.71–3.71)	0.26
TDT-line coverage group (unfavorable vs. favorable)	2.61 (1.13–6.00)	0.02
Recurrent disease (yes vs. no)	1.23 (0.54–2.79)	0.62
TDT-line coverage group (unfavorable vs. favorable)	2.58 (1.12–5.96)	0.03
Pathology (AA/AO vs. GBM)	1.00 (0.45–2.26)	0.99
TDT-line coverage group (unfavorable vs. favorable)	2.54 (1.06–6.07)	0.04
Location (insula, thalamus, corpus callosum vs. lobes)	1.05 (0.42–2.62)	0.91
TDT-line coverage group (unfavorable vs. favorable)	2.06 (0.76–5.59)	0.16
Tumor volume (>10 cm^3^ vs. ≤10 cm^3^)	1.48 (0.59–3.75)	0.41
TDT-line coverage group (unfavorable vs. favorable)	2.91 (1.22–6.97)	0.02
Preoperative KPS (80 or less vs. 90)	2.30 (1.34–3.96)	0.003
TDT-line coverage group (unfavorable vs. favorable)	2.83 (1.17–6.86)	0.02
Preoperative neurologic function status (symptomatic vs. asymptomatic)	1.16 (0.48–2.78)	0.75
TDT-line coverage group (unfavorable vs. favorable)	2.70 (1.00–7.28)	0.05
Intracranial hemorrhage (no vs. yes)	1.65 (0.57–4.81)	0.36
TDT-line coverage group (unfavorable vs. favorable)	3.02 (1.21–7.54)	0.02
Adjuvant treatment (none vs. RT and chemo)	3.33 (1.03–10.79)	0.04
Adjuvant treatment (RT or chemo vs. RT and chemo)	2.08 (079–5.51)	0.14

Analysis of OS produced no statistically significant results, however, the majority of cases (66%) were still being followed at the time of the analysis.

### Other analysis

Postoperative complications were observed in a few patients. In general, these complications were subtle and temporary in the majority of cases where they occurred. Any type of complication was observed after 13/35 LITT procedures (37%). The most common complication was a worsening of preoperative neurological deficit (usually motor) in seven (20%) cases. In five (14%) cases, the new deficit resolved within a few days; however, the new deficit was permanent in two (6%) cases. One patient experienced new seizure, one patient had postoperative hyponatremia and one patient developed bilateral deep vein thrombosis (DVT). Infection was observed in two (6%) cases, one was superficial wound infection in an old patient with history of prior radiation and the other one was ventriculitis.

A small amount of blood products seen within the area of lasing on neuroimaging after LITT treatment was observed in more than half of the patients. This was deemed to be hemorrhagic necrosis that was consistent and appropriate for the case. Distinct from this typical treatment response, in three (8%) cases the amount of hemorrhage was characterized as a moderate to large intra-cerebral hemorrhage (ICH) compared with the initial tumor size. One of these hemorrhages occurred in a patient with multiple other complications—intraventricular hemorrhage from surgery, obstructive hydrocephalus resulting from the hemorrhage, systemic infection unrelated to the procedure itself, and ultimately mortality when the patient's family chose withdrawal of care. No surgical evacuation was performed for any of these cases of ICH due to the deep location of the initial tumors and subsequent hemorrhages. All three of the patients who developed large ICHs harbored newly diagnosed GBM and one of them underwent biopsy on the same day as the LITT.

## Discussion

Despite being used for more than two decades, there is limited published data regarding clinical outcomes from LITT treatment for high-grade glioma patients. The largest series previously published was by Schwarzmaier et al. He reported on the OS results from LITT in 16 patients with recurrent GBM. The reported median survival for these patients was 6.9 months after LITT and was relatively better than the historical control for recurrent GBM patients at that time. No permanent neurological deficit or mortality was reported in that series 14,29. The second largest series of LITT in high-grade glioma was published by Sloan and colleagues. This series was a phase-I, IDE managed study to evaluate safety of the NeuroBlate System in 10 patients with recurrent GBM. Median OS was 10.5 months after the LITT procedure and no mortality was related to the procedure 13. Beyond the two previously mentioned studies, there are a few other case reports and small case series (less than 10 patients each) with less efficient irradiation doses (<6 W) 17,19,25,30. Interestingly, none of the prior studies have published data regarding the PFS of the patients.

We evaluated 24 GBM and 10 anaplastic glioma patients who had undergone LITT as an upfront (16 cases) or salvage treatment (19 cases) for their tumors. Median tumor coverage by the blue and yellow TDT lines was 91% and 98%, respectively. Similarly, median tumor volumes missed by the blue and yellow TDT lines were 0.66 and 0.19 cm^3^. Based on subsequent statistical analysis, a combination of residual tumor volume missed by the yellow TDT line <0.05 cm^3^ plus additional residual tumor volume missed by blue TDT line <1.5 cm^3^ was considered as the TDT-line coverage prognostic factor for PFS (*P* = 0.02). In a one-by-one comparison of the TDT-line prognostic groups with all of the other potential prognostic factors, the effect of the TDT-line prognostic groups remains statistically significant regardless of the factor considered, with the exception of tumor volume. However, in recursive partitioning analysis, at least for the tumors with a volume smaller than 10 cm^3^, the TDT-line prognostic group can differentiate between patients with a better or worse prognosis for PFS (*P* = 0.02).

There are no data in the literature regarding the role of the extent of tumor coverage by the LITT-induced hyperthermic field on outcome for high-grade glioma patients. However, the neurosurgical literature does contain data regarding the role of the extent of open surgical resection on improving outcomes for patients with high-grade gliomas 31. Most of these studies focused on OS rather than PFS 6–8,10. Jeremic et al. 32 in a review of 86 patients with newly diagnosed GBM showed surgical resection versus biopsy has a better outcome in terms of time to progression (33 vs. 21 weeks). Keles and coworkers also reported a better time to progression with increase in the extent of resection in a volumetric analysis of 92 GBM patients 33. Ushio and colleagues reviewed 105 consecutive GBM patients and showed better PFS for patients after complete resection (10 months) compared to partial resection (5 months) and compared to biopsy (3.6 months) 34. Finally, in another report Keles analyzed 102 AA patients and showed that the volume of residual tumor identified on the postoperative T2 MRI sequence has an impact on time to progression of these tumors 9.

In this multicenter study, which is the largest series reported regarding the LITT procedure, we report our experience using the NeuroBlate System for the treatment of high-grade glioma patients with a primary focus on PFS using precise volumetric analysis. As was shown above, near-complete coverage of the tumor by the TDT lines has an impact on enhancing PFS for these patients. A brief review of the existing literature regarding the role of “extent of resection” from open surgical treatment in high-grade gliomas suggests that the same value of “extent of surgical resection” in PFS can be achieved by the “extent of ablation” by the hyperthermic field in the LITT procedure. Hence, the cytoreductive effect of hyperthermia can be considered as equivalent to surgical debulking.

### Limitations

There are several limitations to this study. First, selection bias is an inherent risk for nonrandomized retrospective studies and our study is not an exception. However, every effort was made to try to include all consecutive cases with pathological diagnosis of high-grade glioma which were treated with LITT during the study period at all three centers. Second, despite being a multicenter study, the number of patients is still small, and as a result the study conclusions must be interpreted carefully. Third, because of the short median follow-up period and small number of patients we were not able to determine the effect of TDT-line coverage on OS. Finally, in future reports with larger numbers of patients, inclusion criteria should be limited to only one pathologic tumor type (GBM or AO or AA) with additional consideration of the genetic and micromolecular features of each patient's tumor (e.g., IDH1, MGMT promoter methylation, etc.), especially for OS analysis. Similarly, separate analyses should be done for patients treated with upfront versus salvage procedures.

## Conclusion

Laser interstitial thermal therapy has been shown to have promising results as a safe and effective treatment modality for high-grade glioma patients in conjunction with standard medical and radiation therapies. The prognostic role of the coverage of tumor volume by the hyperthermic field could be considered analogous to the concept of “extent of resection” in surgical treatment of high-grade gliomas. Further prospective studies are in preparation to confirm the effectiveness of LITT for these challenging tumors.

## Conflict of Interest

The following disclose conflicts of interest with Monteris Medical: Tatter—Paid travel expenses to attend a meeting to discuss future research; Barnett—Consultant Medical Director; Equity Interest; Leuthardt and Mohammadi—Consulting. No disclosure for the rest of authors.
